# Endocytosis and Physiology: Insights from Disabled-2 Deficient Mice

**DOI:** 10.3389/fcell.2016.00129

**Published:** 2016-11-25

**Authors:** Wensi Tao, Robert Moore, Elizabeth R. Smith, Xiang-Xi Xu

**Affiliations:** Sylvester Comprehensive Cancer Center and Department of Cell Biology, Graduate Program in Cell and Developmental Biology, University of Miami School of MedicineMiami, FL, USA

**Keywords:** endocytosis, mammary, involution, kidney, adipocyte, liver, cholesterol, LDL

## Abstract

Disabled-2 (Dab2) is a clathrin and cargo binding endocytic adaptor protein, and cell biology studies revealed that Dab2 plays a role in cellular trafficking of a number of transmembrane receptors and signaling proteins. A PTB/PID domain located in the N-terminus of Dab2 binds the NPXY motif(s) present at the cytoplasmic tails of certain transmembrane proteins/receptors. The membrane receptors reported to bind directly to Dab2 include LDL receptor and its family members LRP1 and LRP2 (megalin), growth factor receptors EGFR and FGFR, and the cell adhesion receptor beta1 integrin. Dab2 also serves as an adaptor in signaling pathways. Particularly, Dab2 facilitates the endocytosis of the Ras activating Grb2/Sos1 signaling complex, controls its disassembly, and thereby regulates the Ras/MAPK signaling pathway. Cellular analyses have suggested several diverse functions for the widely expressed proteins, and Dab2 is also considered a tumor suppressor, as loss or reduced expression is found in several cancer types. Dab2 null mutant mice were generated and investigated to determine if the findings from cellular studies might be important and relevant in intact animals. Dab2 conditional knockout mice mediated through a Sox2-Cre transgene have no obvious developmental defects and have a normal life span despite that the Dab2 protein is essentially absent in the mutant mice. The conditional knockout mice were grossly normal, though more recent investigation of the Dab2-deficient mice revealed several phenotypes, which can be accounted for by several previously suggested mechanisms. The studies of mutant mice established that Dab2 plays multiple physiological roles through its endocytic functions and modulation of signal pathways.

## Introduction

Disabled 2 (Dab2) is an endocytic adaptor: Dab2 may impact the uptake of certain extracellular proteins or molecules and may also influence cellular signaling by trafficking components in the pathways. A large number of cellular functions have been proposed for Dab2 and studied in cell culture systems. Although, Dab2 constitutive knockout mice are early embryonic lethal, cre-lox approach enables the study of its functions in physiology. Particularly, Dab2 null mice can be produced by mosaic deletion using Sox2-Cre. Characterization of Dab2 null mice revealed several phenotypes, both expected and surprised, and verified the links between cellular functions in endocytosis and signaling to the identified physiological functions (Table 1).

**Table 1:** **Phenotypes of Dab2 mutant mice**.

**Tissues/cell types**	**Phenotypes**	**References**
Blastocysts/primitive and extraembryonic endoderm early embryos	Failed cell sorting	Morris et al., 2002b; Yang et al., 2002b, 2007; Moore et al., 2013
Kidney/proximal convoluted tubule epithelia	Mild proteinuria	Morris et al., 2002b; Moore et al., 2013
Mammary glands/epithelia	Delayed involution	Tao et al., 2014
Liver/sinusoidal endothelial cells	Increased serum cholesterol	Moore et al., 2013; Tao et al., 2016a
Adipose/Adipocytes/pericytes	Resistant to induced adipogenesis	Tao et al., 2016b
Immune system/Treg	No obvious phenotype *in vivo*; failed to suppress T cells	Jain et al., 2009
Tumors/multiple types	Uterine hyperproliferation, Ovarian epithelial growth, Increased tumor incidence	Yang et al., 2006; Moore et al., 2013

## Dab2 identification and a brief history

Disabled-2 (Dab2), a mammalian ortholog of *Drosophila* Disabled (Gertler et al., 1989), was first isolated from a murine macrophage cell line as a phospho-protein, p96, involved in CSF-1 signal transduction (Xu et al., 1995). The first report of Dab2 in the literature was a short fragment of mRNA identified in differential screening, known as DOC-2 (differentially expressed in ovarian cancer; Mok et al., 1994). At a later time, another Dab2 cDNA was also isolated as a gene induced by castration in rodent models, named as C9 (Tseng et al., 1998).

Sequence alignment indicated that mammalian Dab2 and a neuron specific isoform Dab1 are orthologs of the Drosophila Disabled gene, which was suggested to play a role in neuron development in fly. In mammals, Dab2 is widely expressed (Xu et al., 1998; Fazili et al., 1999), but Dab1 is restricted to brain (Howell et al., 1997; Sheldon et al., 1997). Several spliced isoforms including p96, p93, and p67 were identified as the protein products of the Dab2 gene (Xu et al., 1995; Sheng et al., 2000a, 2001).

Among the protein sequences included in the NBCI database (https://www.ncbi.nlm.nih.gov/protein/?term=Dab2), mammalian Dab2 is most similar to Dab1, Numb, Numbl, and Arh, which are all endocytic adaptor proteins. The homology is highest at the C-terminus, defined by a domain known as PTB/PID (Phospho-Tyrosine Binding or Phosphotyrosine Interacting Domain) domain (Bork and Margolis, 1995).

Dab2 is essential for mouse embryonic development and gene deletion in mice causes early embryonic lethality at a stage prior to gastrulation (Morris et al., 2002b; Yang et al., 2002a, 2007). The embryonic phenotype of the mutant mice indicates that Dab2 is critical in the development of extra-embryonic endoderm. In the *dab2*-deficient embryos, extra-embryonic endoderm cells are present but fail to sort and form primitive and subsequent visceral endoderm epithelia. Rather, the extra-embryonic endoderm cells intermingle with the ectoderm cells (Yang et al., 2002a, 2007, 2009; Moore et al., 2013). Analyses in embryoid bodies and cultured cells support that Dab2 is critical for the surface targeting and restriction of cell adhesion molecules and the establishment and maintenance of epithelial polarity (Rula et al., 2007; Moore et al., 2013). Therefore, Dab2 functions in endocytic trafficking to sustain cell polarity and thus epithelial organization, and hence loss of Dab2 leads to endoderm epithelial disorganization and failure of the embryos (Rula et al., 2007; Yang et al., 2007; Moore et al., 2013).

## Dab2 as an endocytosis adaptor

**Figure 1:**
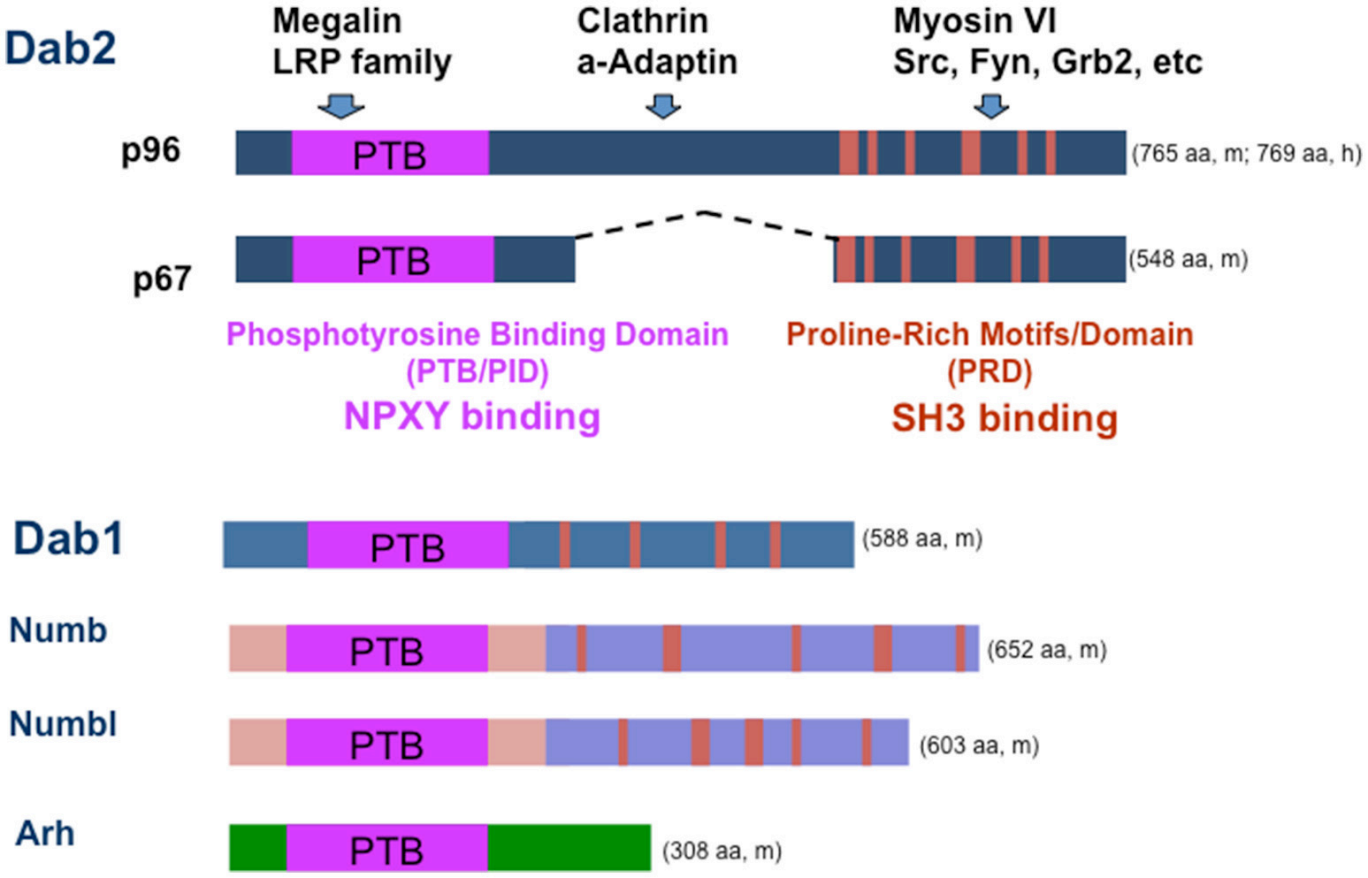
**Structure of Dab2 adaptor and a working model of its function in endocytosis and signaling**. Dab2 has two main spliced forms, p96 and p67. The N-terminal PID/PTB domain of Dab2 binds to the NPXY motif in the intracellular domain of transmembrane glycoproteins/receptors such as LDL receptor, LRP family members, and integrins. The middle and C-terminal portions of Dab2 interact with endocytosis proteins clathrin and alpha-adaptin. The C-terminal portion of the protein also can bind myosin VI, a motor protein that mediates endocytic trafficking. Thus, Dab2 links endocytic vesicles to myosin VI motor trafficking along cellular actin filaments, targeting membrane proteins on the cargos to unique cell surface domains. Dab2 also has a proline-rich domain (PRD) that can bind SH3-containing proteins such as Grb2, Fyn, and Src. Dab2 protein is most similar to other adaptor proteins including neuronal specific family member Dab1, Numb, Numbl, and Arh. The amino acid (aa) numbers are indicated for either mouse (m) or human (h) proteins.

The domains and sequence motifs indicate that Dab2 is an endocytic adaptor protein (Mishra et al., 2002; Bonifacino and Traub, 2003; Hasson, 2003; Figure 1). At the N-terminus, Dab2 possesses a PTB/PID domain that can bind an NPXY (Asn-Pro-X-Tyr, X represents any amino acid) motif found in a subset of cell surface receptors (Bork and Margolis, 1995). Through its PTB domain, Dab2 mediates the attachment of cargos containing transmembrane proteins with an NPXY motif, such as the LDL receptor, EGF receptor, megalin, and integrins, to clathrin coats (through DFF motifs; Mishra et al., 2002; Morris et al., 2002a; Bonifacino and Traub, 2003; Hasson, 2003). Dab2 proteins also contain clathrin binding, NPF, and DPF motifs, which bind components of endocytic vesicles such as clathrin, alpha-adaptin, and EPS-15, respectively (Traub, 2003). The C-terminus binds to the myosin VI motor protein (Inoue et al., 2002; Morris et al., 2002a; Hasson, 2003; Spudich et al., 2007). Thus, Dab2 links clathrin-coated cargos containing transmembrane proteins with an NPXY motif to the myosin motor, facilitating their endocytosis and directional trafficking (Morris et al., 2002a; Hasson, 2003). Studies in cultured cells suggested a role of Dab2 in endocytic recycling and trafficking of integrins and thus impact cell mobility (Caswell et al., 2009; Chao and Kunz, 2009; Chetrit et al., 2009; Teckchandani et al., 2009). Also, participation in the clathrin-mediated endocytosis and trafficking of E-cadherin explains its affects on epithelial organization (Yang et al., 2007). Thus, its functions in mediating polarized trafficking of cell adhesion molecules such as integrins and E-cadherin provide possible mechanisms for failure in endoderm epithelial polarity, sorting, and organization of Dab2 deleted embryos (Yang et al., 2007; Moore et al., 2013).

In addition, Dab2 has feature of an signaling adaptor, as the C-terminal part of the protein contains a proline-rich domain that mediates the association of Dab2 with additional proteins, such as Grb2, Src, and Fyn (Xu et al., 1998; Figure 1). Thus, a scenario is presented that Dab2 also mediates the endocytosis and trafficking of components of various signaling pathways. By doing so, Dab2 acts to modulate cellular signalings. Indeed, endocytosis and trafficking of cell surface receptors and signaling components have been suggested to account for its activities in modulating multiple cellular signaling pathways, including Ras/MAPK (Xu et al., 1998; He et al., 2001; Zhou and Hsieh, 2001), TGF-beta (Hocevar et al., 2001, 2005; Prunier and Howe, 2005; Hannigan et al., 2010), and Wnt (Hocevar et al., 2003; Jiang et al., 2008, 2009, 2012).

The Dab2 protein domains and structure are similar to a family member Dab1 in mammals, though Dab1 is mainly expressed in brain and neuronal cells (Howell et al., 1997). No redundancy between Dab1 and Dab2 activity has been found or reported, likely because of their divergent tissue expression. Dab2 protein sequences are also similar to other signal or endocytosis adaptor proteins including numb, numbl, and Arh protein (Figure 1). Compensatory expression and partial functional redundancy by numb and Arh have been noted in Dab2 knockout mice (Moore et al., 2013; Tao et al., 2014, 2016a).

## Dab2 in the regulation of cellular signaling pathways

In cell culture studies, Dab2 also was shown to modulate several signaling pathways. It was reported that Dab2 serves as an adaptor linking the transforming growth factor beta (TGF-beta) receptors with the Smad family proteins, facilitating TGF-beta signal transduction pathway (Hocevar et al., 2001). Another study indicates that Dab2 facilitates the cellular endocytosis and trafficking of TGF-beta receptor (Penheiter et al., 2010). Additional studies suggested detailed mechanisms for the participation of Dab2 in regulating the TGF-beta pathway (Hocevar et al., 2005; Prunier and Howe, 2005; Chaudhury et al., 2010). In contrast, another study found that Dab2 dose not impact the canonical TGF-beta pathway but Dab2 loss in head and neck cancer compromised the tumor suppressor function of TGF-beta, while enabling its tumor-promoting activities, and concluded that Dab2 is a molecular switch for TGF-beta from a tumor suppressor to a promoter (Hannigan et al., 2010). Thus, several mechanisms have been suggested for the involvement of Dab2 in TGF-beta pathway, such as receptor recycling, adapting function for Smad activation, or non-canonical TGF-beta signaling through the Ras-MAPK pathway, although the actual operation of these mechanisms in intact animals and physiological settings have yet to be demonstrated.

Through its functions in endocytosis of signaling components such as Axin or LRP6, Dab2 is also suggested to plays roles in the regulation of the Wnt pathways (Hocevar et al., 2003; Jiang et al., 2008, 2009, 2012). Deletion of Dab2 in zebrafish embryos through Cas9 mutagenesis resulted in a significant reduction in cardiomyocyte number (Hofsteen et al., 2016), though impact of Dab2 deletion on cardiac development is not notable (Morris et al., 2002b; Moore et al., 2013).

**Figure 2:**
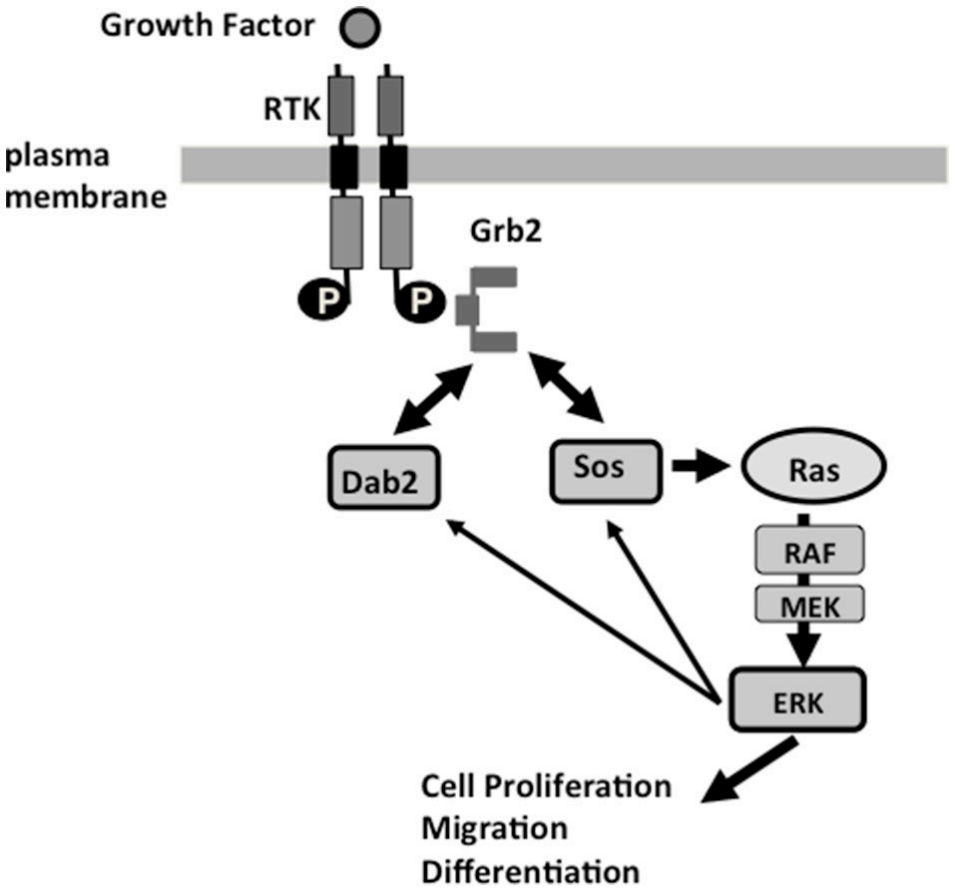
**Dab2 modulation of Ras/MAPK pathway at the endosomal membranes, and Dab2 function in a feedback mechanism of the Ras/MAPK pathway**. Receptor tyrosine kinases (RTK), including EGF and TGF-beta receptors, signal through the Grb2-Sos1 complexes and their activation of Ras, and downstream cascade Raf, MEK, and Erk1/2 to regulate cell proliferation, motility, and differentiation. Grb2 interacts through its SH2 domain binding to phosphotyrosine residue of the activated RTK, and binds via its SH3 domains to the proline-rich domain of Sos1 or Dab2. Phosphorylation of Sos1 and Dab2 by Erk1/2 leads to disassociation of Sos1/2 from Grb2, which is then sequestered by Dab2. The disassembly of Sos1/Grb2 complexes leads to the termination of Ras activation, serving as a feedback regulatory loop in the control of the Ras/MAPK pathway. The re-arrangement of Grb2/Sos1 complex to Grb2/Dab2 association likely coincides with clathrin and adaptor coat disassembly. Loss of Dab2 in cancer cells leads to unsuppressed Ras/MAPK activation.

The Dab2 proline-rich domain (PRD) at C-terminus contains multiple stretches of poly-proline motifs that resemble those in Sos1, a guanine nucleotide exchange factor for and activator of Ras (Xu et al., 1998; Figure 1). Through the PRD, Sos1 binds strongly to the two SH3 domains of the signaling adaptor Grb2, which associates with phosphorylated and activated transmembrane signaling receptors through the SH2 domain of Grb2. Thus, Grb2 recruits Sos1 to membrane receptors to mediate Ras activation (McCormick, 1993). Since the two SH3 domains of Grb2 bind competitively to both Sos1 and Dab2, to their proline-rich motifs, expression levels and phosphorylation of Dab2 and Sos1 modulate the abundance of Sos1/Grb2 complexes. By competing with Sos1 for binding to Grb2, Dab2 acts as a negative regulator for Ras/MAPK pathway (Xu et al., 1998; Zhou and Hsieh, 2001). In cultured cells, Dab2 modulates endosomal Ras/MAPK (Erk1/2) activity by regulating the disassembly of Grb2/Sos1 complexes associated with clathrin-coated vesicles (Xu et al., 1998; Fehrenbacher et al., 2009; Smith et al., submitted). Additionally, a suppressive activity of Dab2 was observed on the MAPK downstream effector, the expression of c-Fos (He et al., 2001; Smith et al., 2001; Yang et al., 2009). The model suggests a role for Dab2 in mediating the endocytosis of signaling complexes and regulating signaling at the endosomes (Figure 2; Vieira et al., 1996). Several events are speculated (Buday et al., 1995; Vieira et al., 1996; Smith et al., submitted): (1) growth factor binds to its tyrosine kinase receptor, which autophosphorylates and recruits Grb2/Sos1 complexes; (2) at the plasma membrane, the activated receptor and signaling complexes initiate the first phase of Ras/MAPK signaling; (3) alpha-adaptin and additional endocytic adaptors such as Dab2 mediate assembly of clathrin-coated pits and the formation of endosomes; 4) the endosome undergoes disassembly of adaptin and clathrin coating, and at the same time, the cargo proteins undergo phosphorylation-triggered rearrangement.

Among the Dab2-associated cellular signaling pathways suggested from cell culture studies, an increase in total Erk1/2 activity was observed in the total embryonic lysate of E9.5 Dab2 null embryos (Smith et al., submitted), suggesting Dab2 has a physiological role in the fine-tuning of the Ras/MAPK pathway. Indeed, a role of Dab2 in suppressing Ras/MAPK activation accounts for embryonic development (Yang et al., 2009) and several physiological phenotypes observed in Dab2 null mice (Tao et al., 2014, 2016b). Probably, the activity as a negative regulator of the Ras/MAPK pathway contributes to the tumor suppressor activity of Dab2 as well.

## Dab2 expression and tissue distribution

Full length Dab2 cDNAs were first isolated from a mouse macrophage cell line (Xu et al., 1995), and a fragment was also identified in another study as a gene that is expressed in human ovarian surface epithelial cells but lost in ovarian carcinomas (Mok et al., 1994). In initial tissue profiling, Dab2 was found widely expressed (Fazili et al., 1999), in contrast to the brain and neuronal specific isoform, Dab1 (Howell et al., 1997).

**Figure 3:**
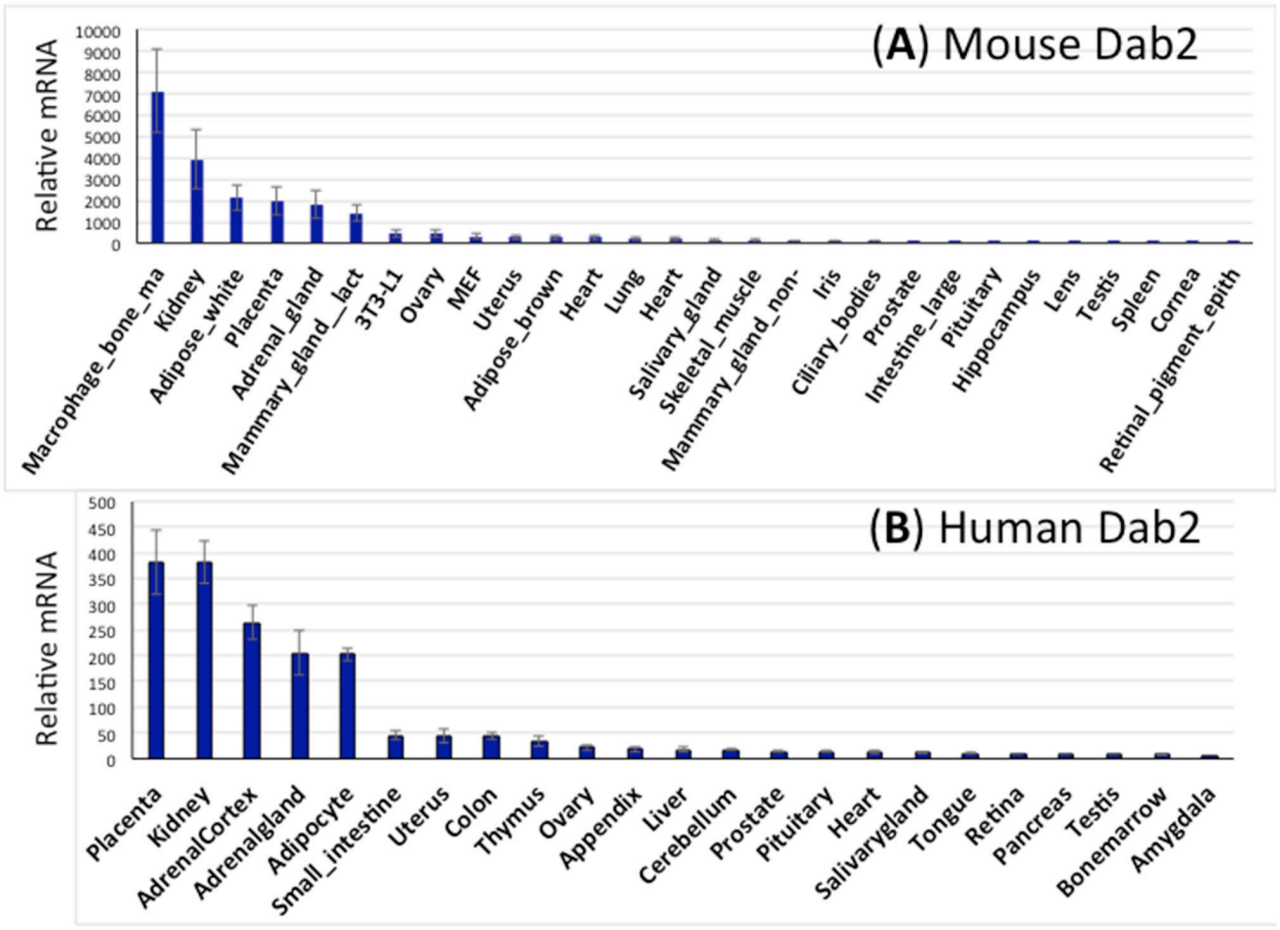
**Expression spectrum of Dab2 mRNA in murine and human tissues. (A)** Dab2 expression in mouse tissues. Microarray data sets were acquired from an online public dataset GeneAtlas MOE430, gcrma (http://biogps.org/#goto=genereport&id=13132). The high-throughput gene expression profiling for Dab2 was generated from a diverse array of normal tissues, organs, and cell lines from mice. The final readouts were averages of four different probe sets with standard error of the mean (SEM) shown as error bars, normalized to housekeeping genes, and ranked according to the abundance of mRNA expression. **(B)** Dab2 expression in human tissues. Similar approaches were used to show Dab2 expression in human tissues.

Using the public GeneAtlas dataset, Dab2 expression is profiled in both mouse (Figure 3A) and human (Figure 3B) tissues. In, Dab2 is seen highly expressed in extraembryonic tissues (placenta) during development. In adults, Dab2 mRNA is present in most tissues at a low level, but is noticeably higher in macrophages, kidney, white adipose tissues, adrenal, and lactating mammary glands (Figure 3). As expected, physiological phenotypes in the Dab2 conditional knockout mice have been observed in many of these tissues or cell types that have high Dab2 expression in wild-type mice (below).

## Function in LDL uptake and cholesterol homeostasis

The members of the LDL (low density lipoprotein) receptor family contain the NPXY motif(s) specifically recognized by Dab2 (Willnow et al., 1999; Herz et al., 2000; Hussain, 2001; Stolt and Bock, 2006), and thus a role in LDL uptake and cholesterol metabolism is anticipated. In studies using cultured cells, Dab2 was found to play a role in the endocytosis of LDL receptor, in coordination with Arh (autosomal recessive hypercholesterolemia; Morris and Cooper, 2001; Maurer and Cooper, 2006). The cellular mechanisms have been investigated and detailed, that Dab2 is able to recruit LDL receptor into clathrin-coated pits and facilitate endocytosis (Maurer and Cooper, 2006; Mulkearns and Cooper, 2012).

Deletion of Dab2 gene in mice only results in a small increase of serum cholesterol level (Moore et al., 2013), while Arh mutation significantly elevates serum LDL and cholesterol levels in mice (Jones et al., 2003, 2007; Harada-Shiba et al., 2004) and also human (Garcia et al., 2001). However, in the absence of Arh, the additional loss of Dab2 greatly augments serum LDL and cholesterol levels to an extent similar to that observed in LDL receptor null mice (Tao et al., 2016a). The finding leads to the conclusion that Dab2 does not cooperate but complements Arh in LDL receptor endocytosis in the clearance of serum LDL particles. Nevertheless, a surprising finding is that Dab2 is not expressed in hepatocytes, but rather in the sinusoidal endothelial cells in liver (Tao et al., 2016a). The study concludes that the combination of Arh and Dab2 is responsible for the majority of adaptor functions in LDLR endocytosis and LDLR-mediated cholesterol homeostasis. In the absence of Arh, Dab2 in liver endothelial cells regulates the expression of HMG-CoA reductase and cholesterol synthesis in hepatocytes (Tao et al., 2016a). How Dab2-mediated endocytosis of LDL in sinusoidal endothelial cells affects cholesterol synthesis in attached hepatocytes has yet to be resolved (Tao et al., 2016a).

## Roles of Dab2 in kidney: protein re-uptake

An initial survey indicated that Dab2 is expressed widely in most tissues and particularly abundantly in the kidney (Fazili et al., 1999), and indeed kidney was confirmed to be one of the highest expressing tissues profiled from a large public expression database (Figure 2), and the expressing cells was found later to be the kidney epithelial cells lining the proximal convoluted tubule (Moore et al., 2013). LRP2/Megalin was one of the first endocytic cargos identified for the Dab2 adaptor (Oleinikov et al., 2000; Yang et al., 2002a), and megalin/Dab2 mediated protein re-uptake in the proximal tubule cells is an likely physiological function of Dab2 in kidney. Indeed, Dab2 null mice have a mild proteinuria (Morris et al., 2002b; Moore et al., 2013). The redundant function of Arh with Dab2 may account for the very mild kidney functional defect in the Dab2 null mice (Nagai et al., 2003).

## Roles of Dab2 in mammary involution

An interest to examine a potential role of Dab2 in mammary glands was prompted by the findings that Dab2 expression is lost in breast cancer (Sheng et al., 2000b) and in carcinogen-induced rodent mammary tumors (Schwahn and Medina, 1998; Medina, 2002). Among the mouse tissues surveyed, the lactating mammary gland has relatively high Dab2 expression (Figure 2). In the general characterization of Dab2 in mice, its expression was found to be absent or very low in virgin mammary epithelial cells, but is greatly induced in mammary glands during pregnancy and lactation (Tao et al., 2014). In primary cultures of mammary epithelial cells isolated from either virgin or pregnant mice, Dab2 expression can be induced by estrogen, progesterone, and prolactin, individually and also synergistically. The hormonal regulation of Dab2 expression in mammary epithelial cells is unique, since the expression was not seen to be significantly altered by sterol hormones in other tissues or cell types.

Based on its functions in endocytosis, initial speculations for the roles of Dab2 in mammary glands during pregnancy and lactation were phenotypes such as cell growth and survival, nutrient uptake, milk production, and secretion that were suggested (Chlon et al., 2008). However, despite rigorous investigation, these phenotypes were not found, and only subtle differences were observed between control and Dab2-deficient mice (Tao et al., 2014). The lack of more noticeable differences in the mammary glands of Dab2 null mice may be due to the compensation of other endocytic adaptors, such as Numb and Arh. Indeed, Numb and Arh protein levels are elevated in Dab2-deficient mammary epithelial cells (Tao et al., 2014).

The most robust phenotype of Dab2 null mammary glands is a transient delay in mammary involution (Tao et al., 2014). For mammary morphology, the impact of Dab2 deletion is most obvious at day 3 of forced involution; however, no permanent changes in mammary glands of Dab2 null mice are noted. The rapid cell death and tissue regression during mammary involution is a tightly controlled process (Furth et al., 1997; Furth, 1999), and TGF-beta signaling is known to play a crucial role (Bierie et al., 2009). In the studies of primary mammary epithelial cells in cultures, Dab2 deletion appears to influence Grb2-mediated TGF-beta Ras/MAPK activation, but does not alter the phosphorylation and activation of the Smad family proteins, which is the canonical pathway of TGF-beta signaling (Tao et al., 2014). Therefore, it was concluded that Dab2 modulates the non-canonical pathways of TGF-beta by suppressing Ras/MAPK activation (Mulder, 2000; Moustakas and Heldin, 2005; Lee et al., 2007; Guo and Wang, 2009; Zhang, 2009; Parvani et al., 2011). The increased TGF-beta-stimulated Ras/MAPK activation and subsequent induction of higher expression of survival genes (Boucher et al., 2000; Balmanno and Cook, 2009) may explain the prolonged survival of mammary epithelial cells of Dab2-null mice during involution (Tao et al., 2014). The role of Dab2 in mammary glands is suggested in that the induction of Dab2 suppresses TGF-beta-induced Erk1/2 activation to allow rapid cell death in mammary involution.

Despite the high expression and hormonal induction of Dab2 in mammary glands of pregnant/lactating mice, only a subtle phenotype (transient delayed mammary involution) was identified in the mammary tissues of the Dab2 null mice (Tao et al., 2014). Induction of Dab2 expression likely acts as a feedback loop for the hormonal induction of mammary gland expansion during pregnancy and lactation, and the presence of Dab2 allows efficient cell death and clearance in mammary involution. From epidemiological studies, pregnancy and lactation at early age is known to have a small protective effect against breast cancer (Kelsey and Gammon, 1991). Thus, the induction of Dab2 expression may contribute to the protection against breast cancer risk that pregnancy and lactation may offer.

## Roles of Dab2 in immune cells

Dab2 cDNA was first cloned from a macrophage cDNA library (Xu et al., 1995), and macrophages are one of the cell types with highest Dab2 expressing (Figure 3). Studies of cultured cells suggested that Dab2 acts in macrophage spreading and adhesion (Rosenbauer et al., 2002). The functions of Dab2 in macrophages were suggested to promote inflammation in brain (Jokubaitis et al., 2013), adipose tissue (Adamson et al., 2016), rat spinal cord injury (Ahn et al., 2011, 2015), and central nervous system (Moon et al., 2005).

Dab2 gene deletion was reported to affect regulatory T cell (Treg) function (Jain et al., 2009). In *in vitro* assay, Dab2-restricted knockout of Treg cells failed to suppress the proliferation of responder T cells. However, mice with Dab2 deletion in Treg cells are healthy in appearance and do not show phenotypes related to autoimmunity defects even at 1 year of age, indicating that the Dab2 is dispensable for Treg cells in maintaining T cell tolerance (Jain et al., 2009). In additional analysis, it was observed that Dab2-deficient Treg cells failed to alleviate established colitis, suggesting a role of Dab2 in regulating lymphocyte infiltration and accumulation in the colon. A mechanism was suggested that the Dab2-deficient Treg cells are poor suppressors *in vitro* due to a deficiency in gap junctions for transferring cAMP to effector T cells, because of a role for Dab2 in endocytic trafficking of connexins, components of the gap junctions (Jain et al., 2009).

Thus, the functions of Dab2 in immunity, either in macrophage or Treg cells, are still an outstanding issue that requires further analyses, especially in challenged conditions.

## Dab2 in tumor suppression

The link of Dab2 to oncogenesis was first made in a differential expression screening experiment, in which a short mRNA fragment was identified as DOC-2 (differentially expressed in ovarian cancer, known as Dab2 at a later time), and the mRNA was found lost in ovarian cancer (Mok et al., 1994). Follow up studies corroborated the loss of Dab2 expression in ovarian and breast cancer and its cell growth suppressive activity, and substantiated the proposal for Dab2 as a tumor suppressor (Mok et al., 1998; Fazili et al., 1999). Another early support was the identification of Dab2 as a suppressed gene in carcinogen-induced mammary tumors in rodents (Schwahn and Medina, 1998). Several subsequent reports corroborated loss or a reduced Dab2 expression in human breast cancer (Sheng et al., 2000b; Bagadi et al., 2007). To date, Dab2 expression has been reported to be reduced or lost in many additional cancer types, including colon (Kleeff et al., 2002), prostate (Tseng et al., 1998), esophageal (Anupam et al., 2006), nasopharyngeal (Tong et al., 2010), and head and neck (Hannigan et al., 2010).

A suggestive observation is that the loss of Dab2 expression occurs in an epithelial layer, and correlates closely with morphological transformation from a benign monolayer to malignant multiple layered ovarian cancer (Yang et al., 2002b). Systematic analysis of a large panel of ovarian carcinomas showed that loss of Dab2 expression occurs in about 90% of ovarian cancer, and is an early event in ovarian tumorigenicity (Fazili et al., 1999).

Earlier analysis of Dab2 tumor predisposable phenotypes was done using heterozygous knockout mice, which were observed to have frequent uterine hyperproliferation and atypia, and increased ovarian epithelial growth and changes (Yang et al., 2006), though no increased tumor incidence was reported in the characterization of a Dab2 conditional knockout line (using Meox2-Cre; Morris et al., 2002b). For another Dab2 conditional line produced more recently, increases in incidence of tumors and preneoplastic lesions were noticed (Moore et al., 2013). The aged Dab2 null mice have higher frequency of tumors upon dissection, observed mainly in the uterus, ovary, liver, mammary gland, and colon, similar to the previous report for the *dab2* heterozygous mice (Yang et al., 2006; Moore et al., 2013). Nevertheless, tumor incidence is relatively low (about 10%) and likely loss of Dab2 contributes but is not sufficiently to cause tumorigenesis.

The tumor suppressor functions of Dab2 have been attributed to its roles in suppressing Ras/MAPK signaling (He et al., 2001; Zhou and Hsieh, 2001), epithelial organization (Sheng et al., 2000b; Yang et al., 2002b, 2007), and cell adhesion/migration (Chetrit et al., 2009; Chao and Kunz, 2009; Chetrit et al., 2009; Teckchandani et al., 2009; Xie et al., 2015).

## Roles of Dab2 in adipocyte differentiation

When Sox2-Cre is used to delete Dab2 gene in the cre-lox conditional knockout mice to bypass the its role in early embryogenesis (Yang et al., 2002a, 2007), live mice are produced (Moore et al., 2013). Sox2-Cre is expressed in the inner cell mass and delete Dab2 gene in a mosaic fashion, and the embryo proper is essentially depleted of Dab2 allele by E9.5 (Moore et al., 2013). Dab2 null mice are produced at an expected ratio and the mice are essentially phenotypically normal in unchallenged conditions (Moore et al., 2013).

**Figure 4:**
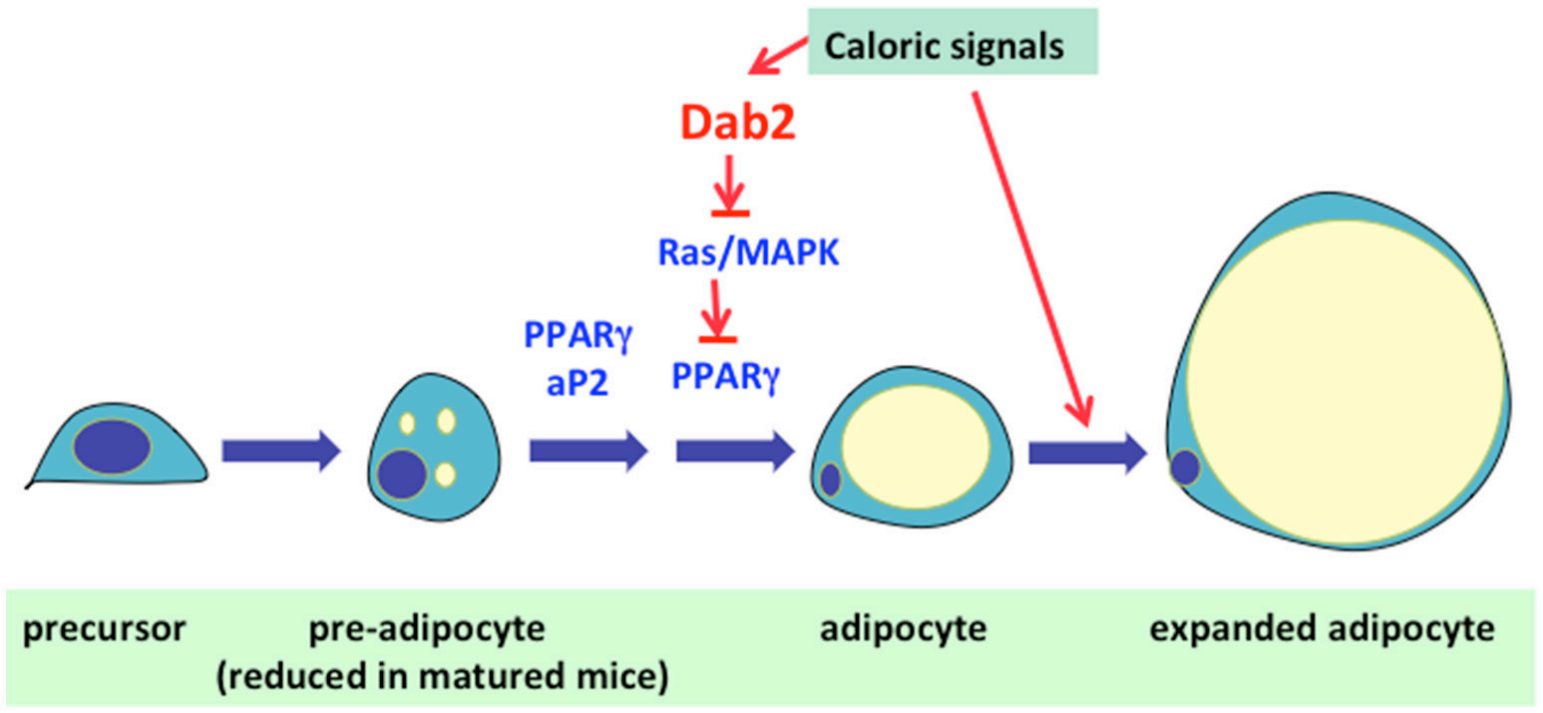
**Role of Dab2 in the regulation of adipocyte cell size and number**. In mammals, excess food intakes/calories are converted into fat (triacylglycerol) and is stored in adipocytes. Expansion of adipose tissue is achieved through either enlargement of cell size or multiplication of cell number. In the first phase, the adipocytes expand in cell size to accommodate the expanding lipid droplet. The second phase of adipose expansion involves the recruitment and differentiation of precursor cells into new adipocytes. The research results suggest Dab2 is essential for the expansion of adipocyte cell number. The preliminary result suggests that Dab2 suppresses MAPK and allows the unsuppressed activity of PPARγ to induce adipocyte differentiation. Thus, unsuppressed Ras/MAPK activation in pre-adipocytes that inhibits the differentiation accounts for the phenotype of resistance to high fat diet-induced weight gain in Dab2 null mice.

In a high fat and caloric feeding experiment that was to investigate cholesterol metabolism, an unexpected observation was that the Dab2 null mice were resistant to high fat diet-induced weight gain (Tao et al., 2016b). The most prominent phenotype of Dab2 knockout mice was their striking lean body composition under a high fat and high caloric diet, although the weight of the mutant mice was indistinguishable from wild-type littermates on a regular chow. The remarkable difference in resistance to high caloric diet-induced weight gain of the *dab2*-deleted mice was present only in juvenile but not in mature mice (Tao et al., 2016b). Restricted Dab2 deletion in adipose lineage using aP2-Cre supports that this phenotype is adipocyte cell autonomous, and the adipose lineage Dab2-deleted (using aP2-Cre) mice have essentially the same levels of serum glucose, lipids, and other metabolites as wildtypes. Based on analyses of cell lines and primary stromal mesenchymal stem cells and cells of the adipose vascular stromal fraction, the mechanism was determined to be a requirement of Dab2 in adipocyte differentiation (Figure 4; Tao et al., 2016b). In the pre-adipocytes, Dab2 is required to suppress the activity of Ras/MAPK, which otherwise phosphorylates the adipogenic master regulator PPARγ and prevents its adipogenic function (Tao et al., 2016b).

Interestingly, the Dab2 null mice are nearly indistinguishable from wildtype mice after birth and when been fed a regular chow diet, suggesting Dab2 is not required for embryonic development of adipose tissues, and also Dab2 is not required for normal function and turnover of adipocytes. Possibly, Dab2 regulates excessive caloric induced pre-adipocyte differentiation, though the idea still requires further analysis and verification. In adipose tissues, Dab2 is found to express in pericytes attached to the vascular structures (Tao et al., 2016b). Whether these Dab2-positive pericytes are pre-adipocytes that can respond to excessive caloric signals will need further investigation.

## Conclusions

The phenotypic characterization of the Dab2 null mice has revealed how endocytosis may impact the physiological functions in intact animals (Table 1). Dab2 is initially expressed and mediates endocytosis in the blastocyst stages, and its function is required for early embryonic development. Although Dab2 is a nonessential gene following completion of development, the absence of the endocytosis adaptor protein impacts diverse physiological functions in whole mice, including cholesterol homeostasis, kidney function, mammary involution, tumor risk, and high calorie-induced adipose expansion, to name the most noticeable few (Table 1).

Based on the cellular study of Dab2 functions in the endocytosis of LRP1 and LRP2 (megalin), integrins, TGF-beta signaling pathway, Wnt pathway, etc, some additional phenotypes are expected, but those are either not found or not significant in whole mice. Redundancy with similar adaptors, especially Arh and numb that are expressed in the same cell types, may obscure the physiological impacts and roles of Dab2. Also, some speculated cellular functions may not be physiological due to low expression in the tissues and cell types and the phenotypes observed in cultured cells do not present in the whole animals.

In sum, although Dab2 null mice are largely normal, slight challenges were able to reveal several physiological changes. Likely, the phenotypes described in this article are incomplete, and surely additional Dab2 physiological functions will continue to be uncovered. So far, the observed physiological functions can be accounted for by the cellular function of Dab2 in endocytosis: substrate uptake and modulation of signaling pathway (especially Ras/MAPK pathway).

## Ethics statement

The experiments using genetic mutant mice were reviewed and approved by the IACUC committee of the University of Miami.

## Author contributions

All the authors have been involved for many years in the area of studies discussed in the article. WT prepared the first draft and XX made extensive revision. ES and RM were also involved in discussion and editing of the article.

### Conflict of interest statement

The authors declare that the research was conducted in the absence of any commercial or financial relationships that could be construed as a potential conflict of interest.

## Abbreviations

AP2: Adaptin protein 2
aP2: adipocyte protein 2
Dab2: Disabled 2
EGF: Epidermal Growth Factor
HMG-CoA: 3-hydroxy-3-methyl-glutaryl-coenzyme A
LDL: low density lipoprotein
LRP: low density lipoprotein-related protein
PRD: proline-rich domain
PTB/PID: PhosphoTyrosine Binding, or Phosphotyrosine Interacting Domain
TGF-beta: transforming growth factor beta
Treg: regulatory T cell.
